# Corneal suture for acute corneal hydrops secondary to post-LASIK ectasia: a case report

**DOI:** 10.5935/0004-2749.20200098

**Published:** 2024-02-11

**Authors:** Daniel Diniz, Fábio Mendonça Xavier Andrade, Wallace Chamon, Norma Allemann

**Affiliations:** 1 Department of Ophthalmology, Escola Paulista de Medicina, Universidade Federal de São Paulo, São Paulo, SP, Brazil

**Keywords:** Dilatation, pathologic, Ectasia, Keratomileusis, laser in situ, Corneal perforation, Hydrops, Keratoconus, Dilatação patológica, Ectasia, Ceratomileuse assistida por excimer laser in situ, Perfuração da córnea, Hidropsia, Ceratocone

## Abstract

Corneal ectasia is one of the main complications of keratorefractive procedures. In this report, we describe a case of corneal ectasia after laser-assisted in situ keratomileusis), which progressed with acute hydrops and aqueous leakage and required a suture for correction.

## Abstract (pt)

A ectasia corneana é uma das principais complicações das cirurgias refrativas. Neste caso, descrevemos um caso de ectasia corneana induzida por *laser-assisted in situ keratomileusis*, que evoluiu com hidrópsia aguda e extravasamento de humor aquoso, necessitando de sutura corneana.

## INTRODUCTION

Laser keratorefractive surgery, also known as laserassisted in situ keratomileusis (LASIK), is the most commonly performed elective surgery in the world.^(1)^ In LASIK, a lamellar corneal flap is created to treat the corneal stroma, using an excimer laser, to reshape the cornea and achieve refractive correction.

Ectasia secondary to keratorefractive surgery is a late-onset complication, characterized by postoperative progressive thinning and protrusion of the cornea. Although the complication rate is low (0.04%-0.6%), the number of cases reported might be underestimated^(2)^. The main risk factors include suspected preoperative topographical abnormalities, residual stromal bed <250 µm, preoperative corneal thickness less than normal, and increased myopia^(3)^. Currently, one of the most important parameters used in preoperative evaluations for LASIK is the percentage of tissue altered (PTA) >40% ([flap thickness + ablation depth]/central corneal thickness)^(2)^.

However, patients who are not considered at risk of developing ectasia also represent a significant challenge for surgeons and require further study.

## CASE REPORT

A 35-year-old female presented with a complaint of decreasing visual acuity in the left eye (oculus sinister [OS]), associated with eye pain, that lasted for a week. She also reported long-lasting, progressive worsening of visual acuity in both eyes. Her medical history included LASIK of both eyes eight years earlier, which had been performed at another service to correct myopic astigmatism. Follow-up showed stability in postoperative years 1-5, but in postoperative year 6, progressive refractive and topographical abnormalities were reported in both eyes.

**Figure 1:**
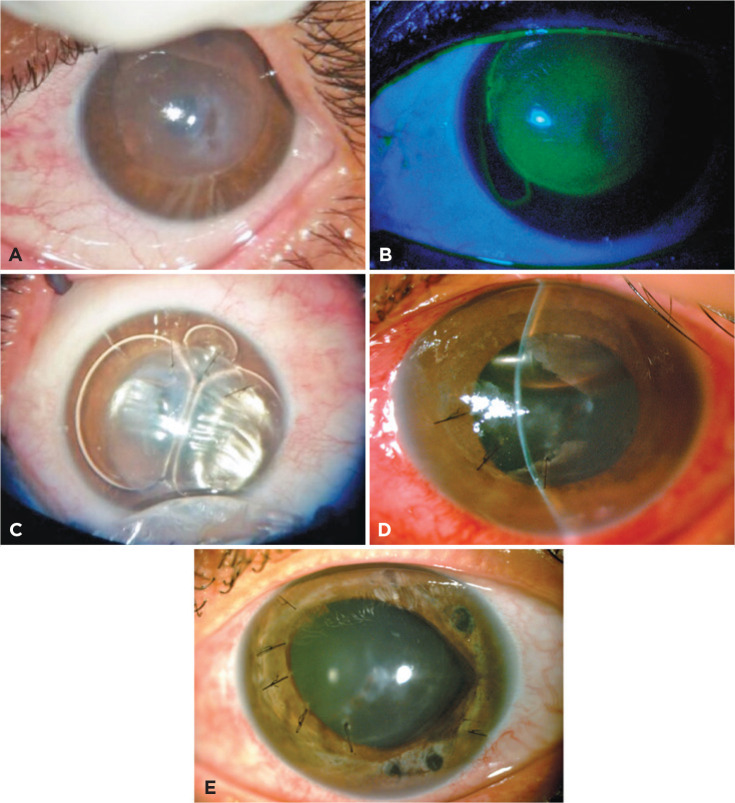
Biomicroscopy of the OS. (A, B) Initial presentation. (A) Central and near-central corneal opacity (stroma edema) with more translucent areas (intrastromal cleft) and hydrops restricted to the LASIK flap. (B) Fluorescein stain, revealing a positive Seidel on the nasal side of the LASIK flap. (C) Intraoperative image after injecting intracameral C_3_F_8_ gas and three sutures on the edge of the LASIK flap, decreasing stromal edema. (D) Ocular pain with angle-closure glaucoma secondary to displacement of gas to the posterior chamber on postoperative day 3, intraocular pressure 55 mmHg, and incomplete mydriasis. Note the decreased central corneal thickness on slit lamp examination. Surgical reintervention was indicated for gas aspiration. (E) Final presentation, 7 days after reintervention, with re-established corneal transparency. Note the sutures on the stromal edge of the LASIK flap and the three iridotomy laser shots at 2, 6, and 12 h. LASIK, laser-assisted in situ keratomileusis.

Ophthalmologic examination showed uncorrected visual acuity of 20/150, corrected visual acuity (after fitting of a gas-permeable contact lens) of 20/50 in the right eye (oculus dextrus [OD]), and hand motions in the OS, whether corrected or uncorrected. Anterior biomicroscopy of the OD showed a transparent lens and cornea, as well as a visible LASIK flap with adequate coaptation. Examination of the OS revealed mild conjunctival hyperemia, central stromal edema, and diffuse corneal opacity, which was suggestive of hydrops. We also found the LASIK flap and more translucent areas in the inferior temporal quadrant (Figure 1A), consistent with intrastromal fluid buildup. The Seidel test result was positive, revealing aqueous leakage through the nasal edge of the LASIK flap (Figure 1B). Applanation tonometry was 18 mmHg in the OD, and digital pressure of the OS showed hypotension. Fundoscopy of the OD was normal, and the OS showed an attached retina, but assessment of details was difficult because of opacity.

**Figure 2:**
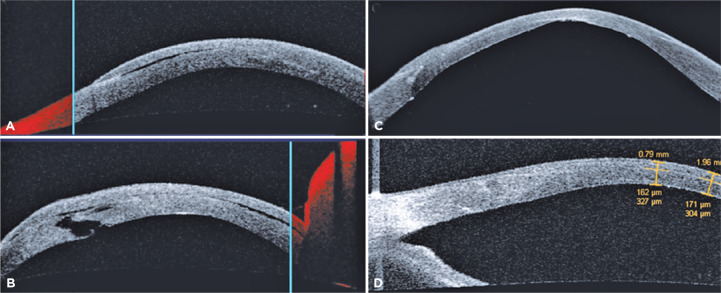
OCT of the OS. (A, B) Initial presentation. Central and near-central corneal hyperreflectivity and thickness consistent with hydrops. (A) Vertical section. Separation of the LASIK flap from the inferior stromal bed (detachment). Central corneal thickness=612 µm. (B) Oblique section. Tear in Descemet’s membrane located inferonasally and associated with intrastromal fluid buildup (fluid-filled cleft). (C) 30-day posthydrop procedure. Vertical section. Reduced central corneal thickness, identified as 262 µm with re-established homogenous reflectivity of corneal stroma. An anterior stromal interface, consistent with a refractive surgery flap, was observed. (D) 1-year posthydrop procedure. Good coaptation of corneal lamellae and surgical incision; corneal thickness=304 µm. OCT, optical coherence tomography; OS, oculus sinister; LASIK, laser-assisted in situ keratomileusis.

Anterior segment optical coherence tomography (AS-OCT) using the Visante OCT system (Zeiss, Oberkochen, Germany) showed that the LASIK flap had separated from the stromal bed, probably because of fluid accumulation (Figure 2A). OCT images at 315° (Figure 2B) revealed a tear in Descemet’s membrane, located in the nasal paracentral area. At this site, increased thickness and stromal hyperreflectivity were consistent with edema, and the tear in Descemet’s membrane was compatible with hyporeflective intrastromal fluid buildup. Overall, these symptoms were compatible with post-LASIK hydrops.

Therefore, 0.1 mL of cyanoacrylate adhesive was applied around the nasal edge of the LASIK flap without raising the flap, followed by placement of a bandage contact lens. The Seidel sign immediately disappeared. However, two days later, the patient experienced increased eye pain. Examination showed no residual adhesive, but spontaneous aqueous leakage had recurred.

The patient underwent surgery to suture the LASIK flap to the stromal bed using three interrupted 10-0 mononylon sutures (Ethicon, NJ, USA), and the anterior chamber was subsequently filled with octafluoropropane (C_3_F_8_) gas (Figure 1C).

However, three days postoperatively, the patient’s eye pain worsened and she experienced headache and vomiting. Examination revealed intense conjunctival hyperemia, apparent sutures in the cornea with properly coapted edges, negative Seidel test results, iris anteriorization, a flat anterior chamber, and displacement of the gas bubble to the posterior chamber (Figure 1D). After the gas moved toward the posterior chamber, there was a complication involving the intracameral gas injection with angle closure and ocular hypertension. We also found a rise in the patient’s intraocular pressure (IOP) to 55 mmHg using a Tono-Pen tonometer (Reichert, Germany).

Therefore, laser peripheral iridotomy was performed, using an yttrium-aluminum-garnet (YAG) laser at three positions, but the patient’s symptoms did not improve. Surgical intervention was required to aspirate the gas bubble, using paracentesis, and the anterior chamber was reconstructed. Thereafter, the patient’s symptoms improved, and the paracentesis sites were sutured. In the immediate postoperative period, the patient’s pain decreased and visual acuity improved.

Postoperatively, on day 7, the patient presented with moderate conjunctival hyperemia; three open iridotomies at positions 2, 6, and 12 h; a wide anterior chamber; and improved corneal transparency (Figure 1E). AS-OCT showed reduced central corneal thickness (262 µm), and the LASIK flap showed adequate coaptation to the stromal bed (Figure 2C).

**Figure 3:**
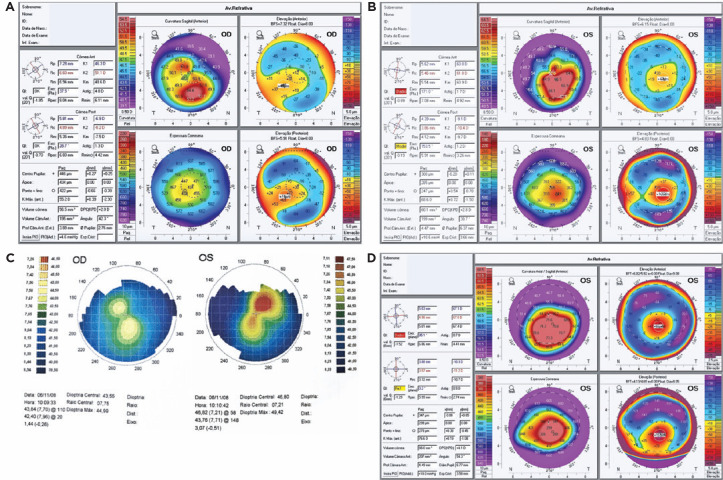
(A) OD. Oblique asymmetrical astigmatism, accentuated inferior near-central toricity. *K*_max_=55.2 D associated with reduced corneal thickness and central pachymetry=446 µm (422 µm at the thinnest point). Post-LASIK ectasia. (B) OS. 30-day posthydrops. Asymmetrical and irregular astigmatism and accentuated inferior near-central toricity. *K*_max_=68.6 D associated with reduced corneal thickness and central pachymetry=300 µm (247 µm at the thinnest point). (**C**) Keratometric maps prior to LASIK with corneal topography (Eyesys). OD. Accentuated corneal toricity and suspected case of ectasia. *K*_max_=44.99 D. OS. Asymmetrical astigmatism, accentuated corneal toricity, and suspected case of ectasia. *K*_max_=49.42 D. (D) OS. 1-year posthydrops. Greater area of asymmetrical and irregular astigmatism and accentuated inferior near-central toxicity. *K*_max_=76.6 D associated with reduced corneal thickness and central pachymetry=347 µm (278 µm at the thinnest point). OD, oculus dextrus; OS, oculus sinister; LASIK, laser-assisted in situ keratomileusis.

During the follow-up examination 30 days postoperatively, we observed improvements in corneal transparency and hyperemia. The OD’s corneal topographic profile showed inferior ectasia with maximum keratometry (*K*_max_) of 55.2 D and pachymetry thinner than 422 µm (Figure 3A). In the OS, we found an area of protrusion with a substantial increase in the inferior curvature, a *K*_max_ of 68.6 D, and pachymetry thinner than 247 µm (Figure 3B). Pre-LASIK corneal topography was available for comparison (Figure 3C). The patient was followed for one year, and her topographic profile presented an increase in *K*_max_ to 76.6 D without a decrease in corneal thickness at the thinnest point (278 µm) (Figure 3D). AS-OCT showed good coaptation of corneal lamellae and surgical incision, with a corneal thickness of 304 µm (Figure 2D).

At this point, we performed adaptation of the rigid gas contact lens. The patient had a visual acuity of 20/100 and was referred for corneal transplantation.

## DISCUSSION

Ectasia is one of the most common post-LASIK complications. In most cases, crosslinking is considered the gold standard to prevent progression^(3)^. Severe cases with acute hydrops in post-LASIK ectasia patients are also common^(2^,^4)^; however, to our knowledge, no studies have discussed aqueous leakage in these cases. Fluid buildup in the flap-stromal bed interface has been reported, and the most common etiology is increased IOP secondary to the use of topical corticosteroids; fluid buildup is also associated with epithelial growth^(5^,^6)^.

Clinical treatment for corneal hydrops includes using hypertonic saline, cycloplegic drugs, and corticosteroids; however, none has a direct impact on resolving the condition. They only provide temporary relief of symptoms until spontaneous resolution occurs^(7)^. Injecting intracameral air or gas is a good option because it decreases contact between the aqueous fluid and the area of the tear in Descemet’s membrane, decreasing stromal edema and accelerating scarring at the site^(8)^.

The surgical approach used in this case was justified by the stromal edema intensity and aqueous leakage. This case is an example of the importance of preand postoperative care for LASIK candidates. It also provides insight into surgical risks in treating acute corneal hydrops, with an emphasis on details of the procedure, including adequate miosis and close follow-up care in the initial postoperative period.
